# Alternative Strategy for Spectral Tuning of Flavin-Binding Fluorescent Proteins

**DOI:** 10.1021/acs.jpcb.2c06475

**Published:** 2023-02-06

**Authors:** Mohammad Pabel Kabir, Daniel Ouedraogo, Yoelvis Orozco-Gonzalez, Giovanni Gadda, Samer Gozem

**Affiliations:** ^†^Department of Chemistry, ^‡^Department of Biology, and ^§^The Center for Diagnostics and Therapeutics, Georgia State University, Atlanta, Georgia 30302, United States

## Abstract

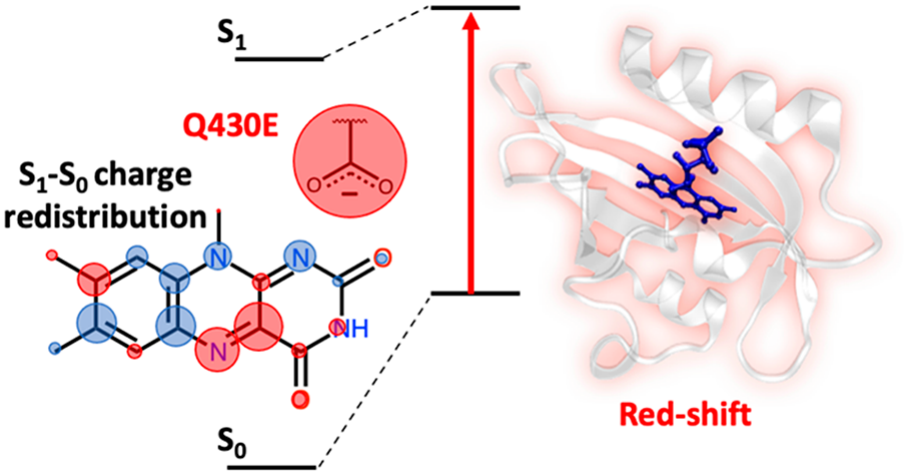

iLOV is an engineered flavin-binding fluorescent protein (FbFP) with applications for *in vivo* cellular imaging. To expand the range of applications of FbFPs for multicolor imaging and FRET-based biosensing, it is desirable to understand how to modify their absorption and emission wavelengths (i.e., through spectral tuning). There is particular interest in developing FbFPs that absorb and emit light at longer wavelengths, which has proven challenging thus far. Existing spectral tuning strategies that do not involve chemical modification of the flavin cofactor have focused on placing positively charged amino acids near flavin’s C4a and N5 atoms. Guided by previously reported electrostatic spectral tunning maps (ESTMs) of the flavin cofactor and by quantum mechanical/molecular mechanical (QM/MM) calculations reported in this work, we suggest an alternative strategy: placing a negatively charged amino acid near flavin’s N1 atom. We predict that a single-point mutant, iLOV-Q430E, has a slightly red-shifted absorption and fluorescence maximum wavelength relative to iLOV. To validate our theoretical prediction, we experimentally expressed and purified iLOV-Q430E and measured its spectral properties. We found that the Q430E mutation results in a slight change in absorption and a 4–8 nm red shift in the fluorescence relative to iLOV, in good agreement with the computational predictions. Molecular dynamics simulations showed that the carboxylate side chain of the glutamate in iLOV-Q430E points away from the flavin cofactor, which leads to a future expectation that further red shifting may be achieved by bringing the side chain closer to the cofactor.

## Introduction

Fluorescent proteins (FPs) have been used as tags for biosensing and bioimaging applications for over two decades in molecular virology and medicine.^1−8^ The most widely used FPs are derived from the green fluorescent protein (GFP), and their spectral properties have been extensively studied experimentally and computationally.^8−16^ However, GFPs have some limitations; they require molecular oxygen and produce hydrogen peroxide during chromophore maturation.^17−19^ They are also ineffective genetic tags in small viruses that cannot handle the genetic load (GFP’s molecular weight is around 22 kDa). For such cases, flavin-binding fluorescent proteins (FbFPs),^20−29^ such as those derived from light-, oxygen-, and voltage-sensing (LOV) domains,^30^ are an attractive alternative because of their smaller size (10 kDa). LOV domains noncovalently bind flavin mononucleotide (FMN), which is readily available *in vivo* and does not require any chemical maturation reaction.^21^ iLOV is a recently engineered FbFP prepared by DNA shuffling of phototropin LOV1 and LOV2 domains.^21,22^ Unlike wild-type LOV domains, iLOV does not contain a cysteine residue near the FMN cofactor and is consequently unable to form the cysteinyl adduct in the excited state that initiates the LOV domain photocycle.

The absorption and fluorescence wavelengths of maximal absorbance of iLOV are around 448 and 500 nm, respectively. iLOV mutants with different colors could be used for multicolor bioimaging and FRET-based biosensing, with several demonstrations already in the literature.^27,31−35^ However, FbFPs are notoriously tricky to spectrally tune without chemical modification of the chromophore. Despite the many mutants expressed, no experimental studies have achieved a larger than 10 nm blue shift in fluorescence emission relative to the original iLOV.^36^ In contrast, attempts to red shift the absorption and emission of FbFPs have met several challenges, as detailed below.

There are two main strategies currently used for the spectral tuning of FbFPs. The first approach is chemical modification of the fluorophore.^37−41^ However, chemically modifying FMN involves synthesizing and loading the chromophore in the protein. This is difficult to achieve *in vivo*, where natural flavin derivatives are instead readily available. The second, more convenient approach would be modulating the natural FMN fluorophore’s electronic energies by modifying the surrounding protein (i.e., by mutagenesis).

**Figure 1:**
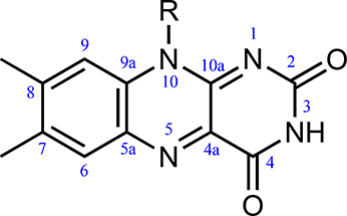
Isoalloxazine ring of FMN and atom number labels. R=CH_3_ for lumiflavin and R = ribose-5′-phosphate for FMN.

Several computational and experimental studies focused on the spectral tuning of iLOV through protein point mutations, with recent attempts primarily focused on red shifting the absorption. Khrenova and co-workers first recognized that placing a positive charge near flavin’s N5 and C4a atoms would red shift iLOV’s absorption and emission wavelength (see Figure 1 for flavin atom labels).^42^ They proposed a Q489K single-point mutation, reasoning that the positively charged amino group (Lys) near flavin’s N5 would stabilize its excited-state π-electron system more than in the ground state. QM/MM calculations supported their hypothesis. However, Davari and co-workers computationally and experimentally showed that the Q489K lysine side chain flips away from the N5 and C4a atoms of the chromophore, resulting in a blue shift in the absorption and emission instead of a red shift.^43^ In a follow-up QM/MM study, Khrenova et al. proposed additional mutations to stabilize the lysine side chain close to the N5 and C4a atoms of the chromophore.^44^ Wehler and co-workers experimentally attempted to prepare these mutants but could not prepare a functional red-shifted FbFP, as they found that a double-point mutant (iLOV-L470T/Q489K) gives a ∼2 nm blue shift and a triple-point mutation (iLOV-V392K/F410V/A426S) lost the ability to bind the chromophore due to the V392K mutation.^45^ Overall, while the strategy of placing a positive charge in the vicinity of flavin’s N5 and C4a atoms was theoretically shown to work, most of the amino acids on that side of the protein turned out to be conformationally unstable or essential for chromophore binding. Recently, Röllen and co-workers prepared a red-shifted iLOV with a double-point mutation (iLOV-V392T/Q489K).^23^ Specifically, while this system showed no shift in absorption, its fluorescence was 6 nm red-shifted relative to iLOV. Red-shifted FbFPs were also derived from the thermostable protein CagFbFP. The largest red shift obtained in this system was 3 nm in absorption and 7 nm in emission with CagFbFP-Q148 K/I52T.^23,46^

Our group recently reported electrostatic spectral tuning maps (ESTMs)^47,48^ and flavin–solvent hydrogen bonding interactions.^49^ Those serve as a starting point to find suitable mutations for spectral tuning. Here, based on these ESTMs, we suggest an alternative mutagenesis approach to red shift the absorption of iLOV; instead of placing a positive charge near C4a or N5, we introduce a negatively charged amino acid in the vicinity of flavin’s N1 atom through a Q430E single-point mutation. We first test the effect of this mutation using hybrid QM/MM calculations employing the average solvent electrostatic configuration (ASEC) free-energy gradient (FEG) approach. We then express iLOV-Q430E in the laboratory and measure its absorption, excitation, and fluorescence spectra to verify if it is red-shifted relative to iLOV.

## Methods

### Computational Approach

The approach to generating ESTMs has already been documented elsewhere^47,48^ and will be briefly summarized in the Results and Discussion section. Here, we focus on the details of the ASEC-FEG calculations. The ASEC-FEG method builds on the average solvent electrostatic configuration (ASEC) approach developed by Canuto and co-workers^50^ and approximates the FEG approach from Okuyama-Yoshida et al.,^51^ which is rooted in the free-energy perturbation theory.^52^ The ASEC-FEG approach was first extended to proteins by Orozco-Gonzalez et al. for rhodopsins.^53^ We recently developed ASEC-FEG for flavoproteins.^54,55^ With ASEC, the quantum chemical calculations are performed in the field of a time-averaged electrostatic potential environment of the protein and solution (collectively referred to as a “solvent” in the ASEC acronym). Effectively, the protein and solution are represented as a superposition of structures obtained from molecular dynamics (MD) simulations. This approach leaves the representation of rigid atoms intact while flexible atoms are replaced by a cloud of charges over the space sampled during the dynamics. Flexible atoms also have a broader and shallower Lennard-Jones potential (see ref (54) for more details). The optimization of the QM system within the ASEC configuration is done self-consistently. ASEC is well suited to averaging the effect of long-range charge interactions.^54^

**Figure 2:**
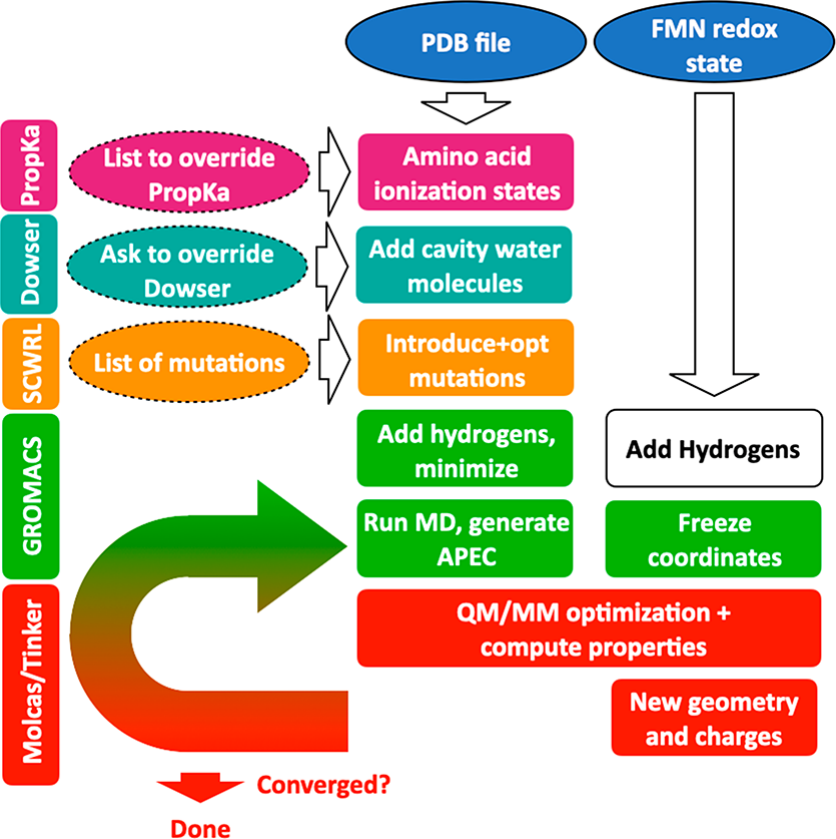
Steps in the automated ASEC-FEG protocol for flavoproteins. Ovals with solid borders indicate required user input, while ovals with dashed borders indicate optional user input.

The ASEC-FEG protocol is a series of scripts building on an existing QM/MM interface between the OpenMolcas^56^ and Tinker^57^ software packages.^58,59^ The protocol guides users through the model construction starting from the PDB file of the protein and culminating in the generation of an ASEC QM/MM model (see Figure 2). The protocol calls on other software: PropKa 3.1,^60^ Dowser,^61^ SCWRL4.0,^62^ and Gromacs.^63^ Gromacs is used to add hydrogen atoms to the PDB, solvate the protein, and run MD simulations to equilibrate the system and sample the protein around the cofactor. The OpenMolcas^56^/Tinker^57^ interface uses an additive QM/MM scheme that includes Lennard-Jones and electrostatic interactions through the electrostatic potential fitted (ESPF) approach.^64^ The automation of this protocol, done in the same vein as efforts to automate the construction of QM/MM models for rhodopsins by Olivucci and co-workers,^65−67^ mitigates problems with the reproducibility of QM/MM calculations and allows the systematic investigation of closely related proteins using a consistent approach.

The initial coordinates of iLOV were taken from the X-ray structure (PDB 4EES, resolution 1.8 Å).^22^ Parameters for FMN were initially retrieved from the AMBER parameter database maintained by the University of Manchester.^68^ The Q430E mutation was introduced into the protein structure and modeled using SCWRL4.^62^ Dowser was used to remove nonbonded water molecules from the crystallographic structure.^61^ The total charge of the systems was neutralized by adding solution counterions. This model was used as a starting point for MD simulations and the generation of the ASEC environment. The MD calculations for iLOV and iLOV-Q430E were performed with periodic boundary conditions in a 7.0 nm × 7.0 nm × 7.0 nm cubic solvent box. The particle-mesh Ewald method with a distance cutoff of 1.2 nm was used. Geometry minimization and MD simulations were carried out using GROMACS.^63^ The AMBER99SB^69,70^ and TIP3P^71^ force fields were used for protein and water, respectively. During each step of the ASEC-FEG cycle, the MD calculations were performed in three phases: the system was first gradually heated from 0 to 300 K at 1 atm pressure over 300 ps. This was followed by 4700 ps of equilibration and 5000 ps of production simulations carried out with the NPT ensemble under standard ambient temperature and pressure. The ASEC configuration of the protein was formed by sampling 100 configurations selected at 50 ps time intervals from the production part of the MD. The ASEC GROMACS file was then converted to Tinker format for QM/MM calculations.

**Figure 3:**
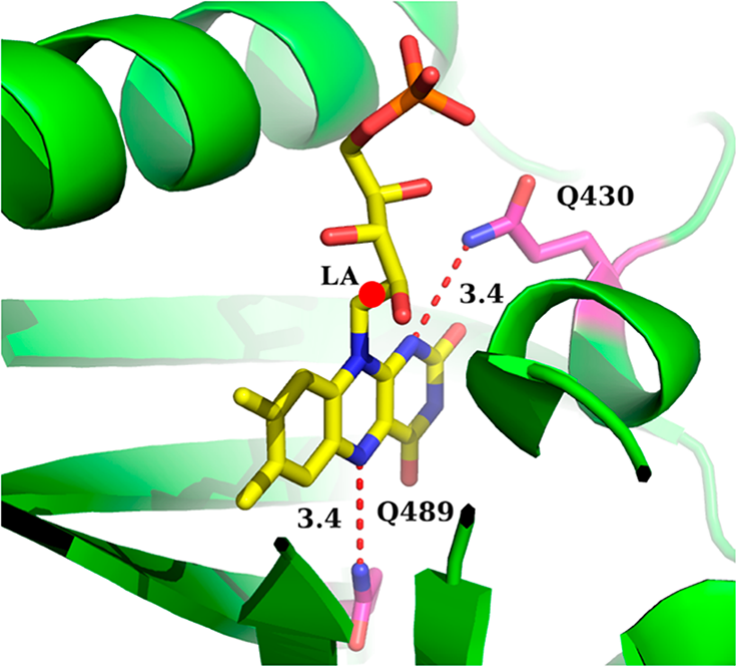
FMN inside the binding pocket of iLOV from PDB 4EES. The dashed lines indicate the distance in angstroms between the Q489 and Q430 glutamine side chain nitrogen atoms and the closest FMN nitrogen atom. The red circle indicates the hydrogen link atom (LA), which separates the QM subsystem (the lumiflavin) and the MM subsystem (the ribose-5′-phosphate group, protein, and solvent). The figure was prepared using PyMol.^72^

For QM/MM calculations, the protein was divided into two subsystems (Figure 3): (i) the QM region, comprising the lumiflavin (structure shown in Figure 1), and (ii) the MM region, which includes all other atoms in the simulation (the ribose-5′-phosphate group, the protein, the solvent, and solution ions). The frontier between the QM and the MM parts is treated using a hydrogen link atom (LA, Figure 3). The charges for the MM atoms near the LA are set to zero and distributed over other MM atoms to avoid overpolarizing the QM wave function. The QM subsystem is then optimized in the presence of a frozen ASEC MM environment with electrostatic embedding. Using the updated geometry and updated ESPF charges of the QM subsystem, another MD calculation is run for 5 ns to generate a new ASEC configuration. This process is repeated for several steps until the computed excitation energies stay consistent for four consecutive steps (i.e., within 0.02 eV nm of the four-step moving average). We then took the average excitation energies from those four steps and used them to compute the wavelength shift.

The geometry optimization of the QM subsystem was performed using the complete-active-space self-consistent field (CASSCF) level of theory and the ANO-L-VDZP basis set. We tested the effect of the active space on the first excited state (π–π*) excitation energy of lumiflavin using gas-phase benchmark calculations shown in Figure S1 in the Supporting Information (SI); we found that there is a limited benefit to increasing the active space beyond 10 electrons and 10 orbitals (5π, 5π*). Therefore, QM/MM geometry optimizations were performed using CASSCF (10, 10). The active space orbitals are shown in Figure S2 in the SI. State averaging was not used for ground-state optimizations, while 2-root state averaging was used for excited-state optimizations. Excitation energies were computed using the complete-active-space second-order perturbation theory (CASPT2) with the ANO-L-VDZP basis set and 8-root state averaging. CASPT2 calculations were performed using the Cholesky decomposition^73^ and applying an imaginary level shift^74^ of 0.2. An IPEA shift,^75^ sometimes used for flavins for the purpose of error cancellation,^76,77^ was not used here. It was recently shown that the IPEA shift is not needed in cases where the dynamical electron correlation is adequately accounted for in CASPT2 calculations.^78^ We also note that vertical excitation energy calculations typically underestimate the absorption wavelength of flavins compared to the experimental wavelength of maximal absorption; the calculation of the vibronic progression from Franck–Condon factors is needed for better quantitative agreement between theory and experimental spectra.^48,79−81^

### Data and Code Availability

The ASEC-FEG protocol is available in an online repository (https://github.com/sgozem/New_APEC_OpenSource). The protocol uses a PDB file as input. All other input files for Gromacs, Tinker, OpenMolcas, SCWRL, and Dowser are included in the code above. The final QM/MM Tinker xyz and OpenMolcas CASPT2 output files are included in the SI.

### Bacterial Strains and Plasmids

iLOV and iLOV-Q430E were obtained as synthetic genes from GenScript Inc. (Piscataway, NJ, USA) and were flanked with 5′-NdeI and 3′-XhoI restriction endonuclease recognition sites in a pET20b(+) plasmid. The pET plasmid harboring the iLOV and iLOV-Q430E genes contains an N-terminal His_6_ tag fused to the target proteins to facilitate heterologous expression in *E. coli* and affinity chromatography purification. The genes were transformed into *E. coli* strain DH5α and Rosetta(DE3)pLysS competent cells for storage and expression, respectively. The resulting plasmids were verified by sequencing (Psomagen, Inc., Rockville, MD, USA), and permanent stocks of the cells were prepared and stored at −80 °C.

### Protein Expression and Purification

Permanently frozen stocks of *E. coli* cells Rosetta(DE3)pLysS harboring iLOV or iLOV-Q430E genes were used to inoculate 100 mL of Luria–Bertani broth medium containing 100 μg/mL ampicillin and 34 μg/mL chloramphenicol, and cultures were grown at 37 °C overnight to be used as a preculture. A 10 mL portion of preculture was used to inoculate 1.0 L of Luria–Bertani broth medium containing 100 μg/mL ampicillin and 34 μg/mL chloramphenicol. When the cultures reached optical densities of ∼0.6 at 600 nm, the temperature was lowered to 18 °C and isopropyl-thio-galactoside (IPTG) was added to a final concentration of 0.1 mM. After 18 h, the cells were harvested by centrifugation at 5000*g* for 20 min at 4 °C. All purification steps were carried out at 4 °C. The wet cell paste was suspended in 0.1 mM PMSF, 0.2 mg/mL lysozyme, 10% glycerol, and 50 mM pH 8.0 phosphate buffer solution containing 300 mM NaCl, 10 mM imidazole, and 10% glycerol in a ratio of 1 g of wet cell paste to 4 mL of lysis buffer. The suspended cells were then allowed to incubate with stirring for 30 min on ice with 5 μg/mL RNase and 5 μg/mL DNase in the presence of 10 mM MgCl_2_. The resulting slurry was sonicated for 60 cycles of 20 s with the pulse on and 10 s with the pulse off for 20 min. The cell debris was removed by centrifugation at 10 000*g* for 20 min. The supernatant was loaded onto a 5 mL Ni-NTA column (GE Healthcare), equilibrated with 50 mM pH 8.0 phosphate buffer solution, 300 mM NaCl, 10 mM imidazole, and 10% glycerol. The proteins were purified with gradient elution from 10 to 250 mM imidazole in 50 mM pH 8.0 phosphate buffer solution, 300 mM NaCl, and 10% glycerol buffer. The eluted fractions containing the iLOV protein were dialyzed against four changes of 10 mM pH 8.0 phosphate buffer solution, 10 mM NaCl, and 10% glycerol. After the dialysis, the proteins were centrifuged at 10 000*g* for 20 min to remove any precipitated protein. The iLOV and the iLOV-Q430E were then stored at −20 °C.

### Flavin Reconstitution

After column chromatography, the iLOV-Q430E variant protein was devoid of bound FMN cofactor. The variant protein was incubated with excess free FMN to load the FMN cofactor into the protein. The free FMN was extracted from the FMN-dependent *Pseudomonas aeruginosa* nitronate monooxygenase (*Pa*NMO) variant, H183F. The incubation was carried out at 4 °C overnight. Amicon Ultra-0.5 centrifugal filters with a 3 kDa molecular weight cutoff were used to remove any free FMN. The samples were centrifuged for several cycles of 8–10 min each until the flow-through was cleared of free FMN. This was verified by taking a UV–visible spectrum of the flow-through, which showed no flavin peaks. Moreover, to ensure that there was no FMN leakage from the holoprotein, extra buffer was added, and the samples were centrifuged again for several cycles. The flow-through gave an identical spectrum, indicating again that it is free of FMN.

### UV–Visible Absorption and Fluorescence Spectroscopy

The UV–visible absorption spectra of iLOV and iLOV-Q430E were recorded with an Agilent Technologies model HP 8453 PC diode-array spectrophotometer equipped with a thermostated water bath. The proteins were prepared fresh by gel filtration through PD-10 desalting columns (General Electric, Fairfield, CT) just before being used. The extinction coefficients of the enzyme-bound FMN to the iLOV protein were determined in 20 mM pH 7.0 phosphate buffer solution after incubation of the protein with 4 M urea at 40 °C for 1 h, based upon an ε450 value of 12.2 mM^–1^ cm^–1^ for free FMN and the method published by Whitby et al.^82^ The fluorescence emission spectra of the iLOV and iLOV-Q430E variant protein were recorded in 20 mM pH 8.0 phosphate buffer solution at 15 °C with a Shimadzu model RF-5301 PC spectrofluorometer using a 1 cm path length quartz cuvette. All fluorescence spectra were corrected by subtracting the corresponding blanks to account for Rayleigh and Raman scattering. The samples at a concentration of 10 μM protein-bound flavin were excited at the low-energy peak of the UV–visible absorption spectrum, and emission scans were determined from 475 to 600 nm.

## Results and Discussion

### ESTMs and Charge Analysis

**Figure 4:**
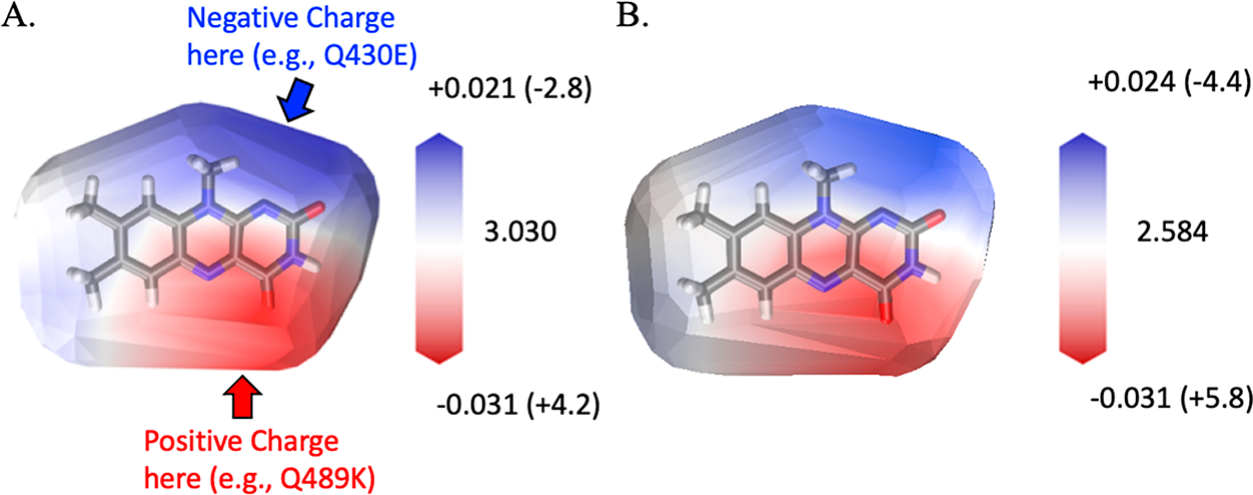
(A) ESTM map for flavin’s first singlet excited state. The map indicates the change in the vertical excitation energy between the ground state (S_0_) and the first singlet excited state (S_1_) introduced by a +0.1 probe charge placed at the van der Waals surface of lumiflavin in its S_0_ equilibrium geometry. The map suggests mutations that red shift the excitation energy (see arrows and labels). The legend indicates the magnitude of the excitation energy shifts relative to the gas-phase reference excitation energy in eV (and in nm in parentheses). (B) The same ESTM map was computed at the S_1_-optimized lumiflavin geometry, corresponding to the fluorescent minimum. The legend indicates the magnitude of the emission energy shifts relative to the gas-phase reference emission energy in eV (and in nm in parentheses). Panel A was partially adapted from ref (47). Copyright 2019 American Chemical Society

ESTMs for flavin were reported recently.^47,48^ These maps are intuitive visual tools that indicate how external positive or negative charges in the vicinity of a molecule influence its absorption or emission spectra. Briefly, ESTMs are constructed by moving a point charge on the van der Waals surface of the molecule and calculating the change in excitation and emission energies. The ESTM for the first singlet excited state (S_1_) of flavin, which is experimentally at ∼448 nm in iLOV, is shown in Figure 4A. Here, we also recomputed the ESTM at the excited-state optimized flavin geometry to map how the fluorescence energy, which is experimentally at ∼500 nm in iLOV, is modified by nearby point charges (Figure 4B). Both ESTMs were computed using time-dependent density functional theory (TD-DFT) with the B3LYP functional^83^ and cc-pVTZ basis set.^84^

The red- and blue-colored regions of the ESTMs in Figure 4 indicate that a spectral shift can be achieved if there is an electrostatic potential change in those regions. Panels A and B of Figure 4 have similar features: a red region near C4a and N5 atoms, a blue region near the N1 atom, and a white region near the xylene portion of flavin. This indicates that (a) a positive charge near the C4a or N5 atoms would red shift the absorption/emission, (b) a positive charge near N1 would blue shift the absorption/emission, and (c) charges introduced near the xylene portion of the flavin would have a negligible effect on the spectral properties for this state. A negative probe charge would have the exact opposite effect.^47^ These calculations are largely consistent with transition dipole moment measurements in flavins.^85,86^ While the excitation energy ESTM (Figure 4A) and emission ESTM (Figure 4B) are very similar, the magnitude of the shift reported in the plot legends reveals a subtle difference: the emission energy (in eV) is more sensitive to the presence of negative charges near the N1 flavin atom than the corresponding absorption energy.

The ESTMs in Figure 4 were used to generate strategies for spectrally tuning lumiflavin. The maps indicate that a positive charge near the C4a or N5 flavin atoms would lead to a red-shifted absorption/emission; this has been the strategy proposed by Khrenova et al. and the ensuing computational and experimental work.^23,41−45^ A second strategy, not yet explored in FbFPs, would be to introduce a negatively charged amino acid in the vicinity of the N1 atom of flavin. Here, we pursue this strategy with Q430E.

**Figure 5:**
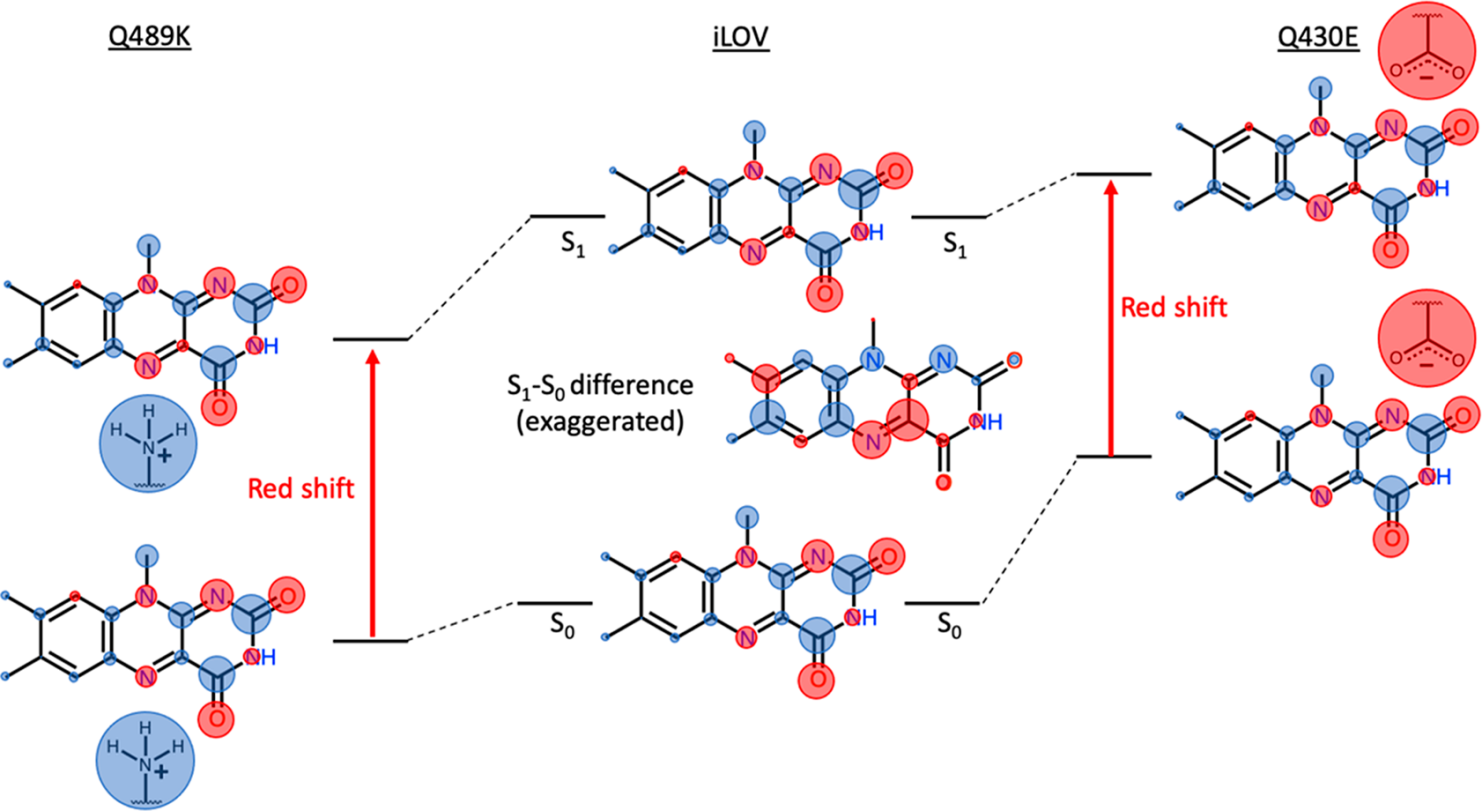
(Center) LoProp charge population analysis (with hydrogen atom charges summed onto the heavy atoms to which they are connected) for lumiflavin in its ground (S_0_) and first singlet excited (S_1_) states. Red circles indicate negative charge density, and blue circles indicate positive charge density. The area of the circles is directly proportional to the charge on the corresponding atom. The S_1_ and S_0_ states have slightly different charge distributions that are difficult to discern without close inspection. Therefore, the difference in the atomic charges (S_1_ – S_0_) is also shown in the middle. In the S_1_ – S_0_ difference plot, the areas of the circles are proportional to the magnitude of the charge difference between S_0_ and S_1_, with red circles indicating reduced charge (higher electron density) on the atom after excitation from S_0_ to S_1_ and blue circles indicating increased charge (lower electron density). (Left) Scheme illustrating the effect of placing a positively charged lysine side chain close to the flavin N5/C4a, which would result in a red shift (see the text for details). (Right) Scheme illustrating the effect of placing a negatively charged glutamate side chain close to the flavin N1, which should also result in a red shift (see the text for details).

To better understand the electronic structure changes underlying the electrostatic spectral tuning properties of flavin, we computed atomic charges from both the ground and excited-state wave functions of a lumiflavin gas-phase model (Figure 5). The charges were obtained using LoProp population analysis from CASSCF(10,10)/ANO-L-VDZP wave functions. The LoProp approach provides physically meaningful localized properties and mitigates issues such as the basis set dependence sometimes encountered with other population analysis methods.^87^

Exciting flavin from the S_0_ to the S_1_ state changes the electron distribution along the π-conjugated isoalloxazine ring. This effect is subtle but can be best visualized by plotting the change in the charges at each atomic center from S_0_ to S_1_ (Figure 5, center). Specifically, there is an increase in electron density at the C4a and N5 flavin atoms and a decrease in electron density at the N1 flavin atom and several atoms in the xylene moiety. There is also a slight decrease in electron density at the C2=O carbonyl. The charge redistribution is consistent with the ESTMs in Figure 4; the increased charge density at the C4a/N5 flavin atoms means there is potential for spectral tuning by placing a positive charge nearby. Such a positive charge, e.g., a protonated lysine side chain, would stabilize the excited S_1_ state slightly more than the ground S_0_ state, leading to a red shift in the excitation energy (Figure 5, left). Conversely, there are several atoms where the electron density decreases upon excitation to S_1_. In most cases, those atoms are shielded from external charges by methyl groups or hydrogen atoms, which explains the less intense color of the ESTM map near the C7, C9, C9a, and N10 atoms despite the decrease in the electron density on those atoms. However, the N1 atom is exposed, allowing charged amino acids to approach and creating an opportunity for spectral tuning at that site. The decreased electron density on the N1 flavin atom means that a negative charge nearby, e.g., a glutamate side chain, would destabilize the ground state more than the excited state, decreasing the S_0_ – S_1_ energy gap and resulting in a red-shifted absorption (Figure 5 right).

The approach shown on the right of Figure 5 (placing a negatively charged amino acid near flavin’s N1 atom) seems less desirable than the approach on the left (placing a positively charged amino acid near flavin’s C4a/N5 atoms) from a bioengineering standpoint since it relies on an unfavorable interaction between flavin and a negatively charged residue. However, given the limited success with spectral tuning at the C4a/N5 site, we attempted spectral tuning with a negatively charged residue with the Q430E single-point mutation.

### QM/MM Simulations of iLOV and iLOV-Q430E

The crystal structure of iLOV indicates that Q430 is 3.4 Å away from the N1 atom of FMN (Figure 3). Therefore, we chose to replace Q430 with isosteric glutamic acid. Since glutamic acid has a p*K*_a_ of 4.07 in solution and Q430 has polar residues nearby, we anticipated that the mutated glutamic acid Q430E would be deprotonated and introduce a negative charge near the flavin’s N1 atom without causing a significant structural change in the protein. To test this hypothesis, we performed QM/MM geometry optimizations followed by excited-state energy calculations for both iLOV and iLOV-Q430E using the ASEC-FEG method, as outlined in the Methods section.

**Figure 6:**
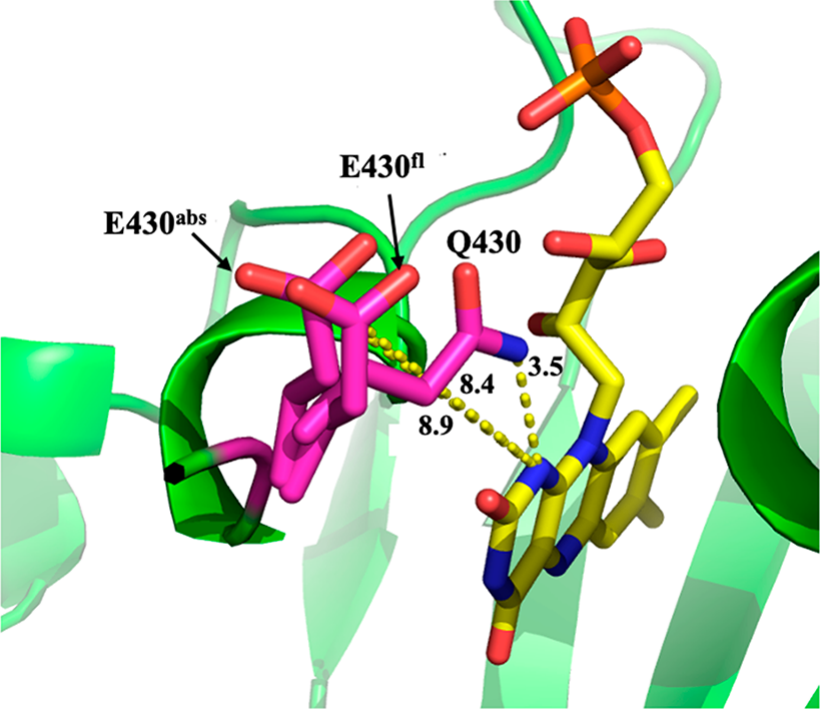
Three representative QM/MM optimized snapshots to show the distance between the flavin N1 and the Q430 (iLOV) or E430 (iLOV-Q430E) side chain. The labeled distances are reported averages from one of the production runs of the MD simulations. Specifically, we label the distance between the flavin N1 and the Q430 side-chain nitrogen in iLOV (3.5 Å), the E430 side-chain carboxylate carbon in the ground-state optimized system (E430^abs^, 8.9 Å), and the E430 side-chain carboxylate carbon in the excited-state optimized system (E430^fl^, 8.4 Å). The figure was prepared using PyMol.^72^

In snapshots obtained from MD simulations of iLOV, the average computed distance of the Q430 nitrogen from the flavin N1 atom (3.5 Å, Figure 6) is in good agreement with the crystal structure (3.4 Å, Figure 3). In contrast, MD simulations of iLOV-Q430E revealed that the E430 glutamate side chain flips away from flavin and maintains an average distance of 8.9 Å from the flavin N1 atom (E430^abs^ in Figure 6). This conformational change is likely driven by a lack of hydrogen bonding with neighboring amino acids, which causes E430 to point outward toward the surface of the protein.

Next, we proceeded to calculate the vertical excitation energy. The iLOV and iLOV-Q430E calculations reached self-consistency quickly after just one step of the ASEC-FEG cycle. After that, the following four steps, each involving MDs to regenerate the ASEC environment, reoptimizing the flavin chromophore, and recomputing the vertical excitation energy, yielded similar results within 1 nm of each other. These calculations indicated that the vertical excitation energy of iLOV-Q430E is 4 nm red-shifted compared to iLOV (Table S1).

In a recent joint computational and experimental study,^54^ we found that solution ions may affect the outcome of ASEC-FEG calculations, especially when there are charged amino acids inside the active site of a flavoprotein. Therefore, we repeated the calculations for both iLOV and iLOV-Q430E after adding 4 pairs of Na^+^ and Cl^–^ solution ions, approximately 1 NaCl per 2775 water solvent molecules, equivalent to 20 mM salt used in the experiments in this work. These calculations showed that the Q430E mutation has almost no effect on the absorption spectrum of iLOV, causing a shift of less than 0.5 nm (Table S2).

To determine if the cause of the red shift is steric or electrostatic, we extracted the lumiflavin geometry from the last ASEC iteration of the iLOV and iLOV-Q430E QM/MM calculations and ran TD-DFT calculations for those structures *in vacuo* using the B3LYP functional and cc-pVTZ basis set. The lumiflavin structure from iLOV-Q430E was found to give a 0.05 eV blue-shifted absorption relative to iLOV. This means that the red-shifted absorption iLOV-Q430E must be due to electrostatic interactions with the protein, as originally suggested, and is not geometric in origin.

The MD and ASEC-FEG simulations show that the E430 does not remain in the same position as Q430 and causes just a slight 0–4 nm shift in the vertical excitation energy relative to iLOV, which was initially discouraging. However, given the difficulty in red-shifting iLOV even by a few nanometers, we decided to proceed with expressing iLOV-Q430E and iLOV experimentally to compare their absorption and emission properties.

### Experimental Absorption and Emission Spectra of iLOV and iLOV-Q430E

**Figure 7:**
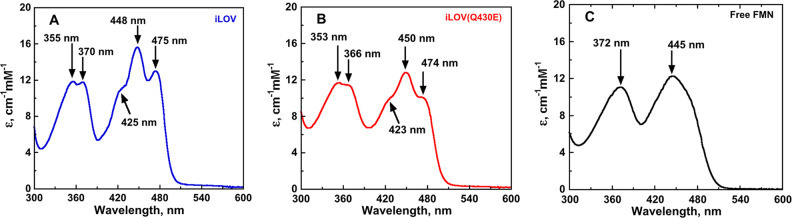
UV–visible absorption spectra of iLOV (panel A, blue), iLOV-Q430E (panel B, red), and free FMN (panel C, black). The spectra were recorded in 20 mM pH 7.0 phosphate buffer solution at 15 °C.

**Figure 8:**
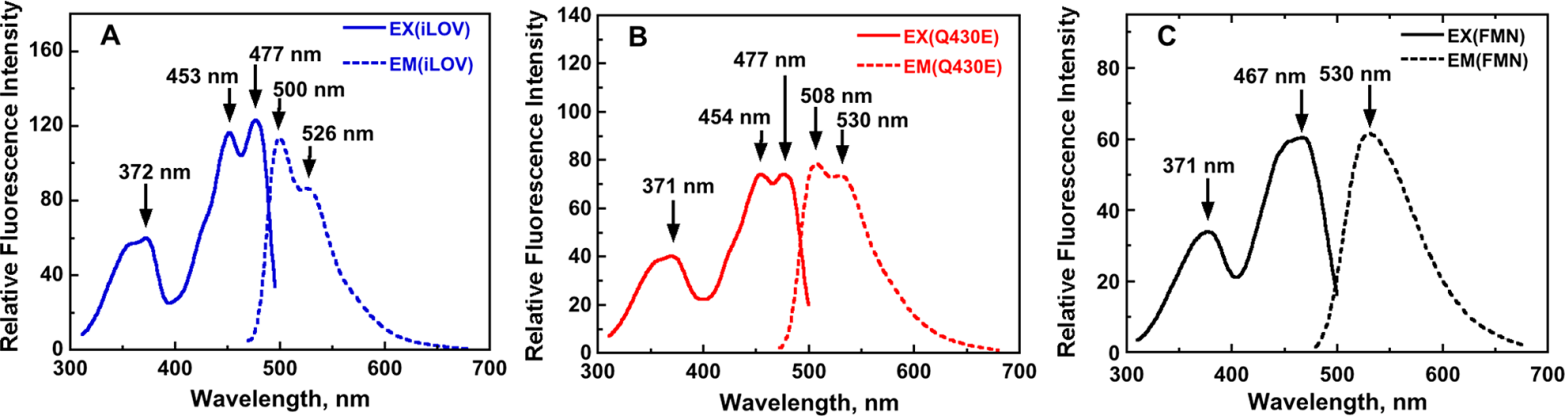
Excitation and emission spectra of iLOV (panel A, blue), iLOV-Q430E (panel B, red), and free FMN (panel C, black). The spectra were recorded in 20 mM pH 7.0 phosphate buffer at 15 °C.

iLOV and iLOV-Q430E were expressed and purified successfully. The absorption and excitation/emission spectra were recorded and are shown in Figures 7 and 8, respectively. The data are also tabulated in Table 1. iLOV-Q430E gave a modest 1–2 nm red shift in the first excited state absorption wavelength, consistent with our computational prediction of a weak red shift. However, the fluorescence wavelength is shifted to the red by 4–8 nm compared to the emission in iLOV. This is comparable to the shift recently achieved by Röllen and co-workers in iLOV-V392T/Q489K (6 nm red shift in emission but no shift in absorption) and in CagFbFP-Q148K/I52T (3 nm red shift in absorption and 7 nm red shift in emission).^23^

**Table 1:** Experimental Absorption and Fluorescence Properties of iLOV Proteins^a^

	iLOV	iLOV-Q430E	free FMN
maximal absorbance,^b^ nm	370, 448	366, 450	372, 445
extinction coefficient,^b^ mM^–1^ cm^–1^	11.9, 15.6	11.8, 12.8	10.0, 12.5
fluorescence wavelength,^c^^,^^d^ nm	500 ± 1, 526 ± 1	508 ± 1, 530 ± 1	530 ± 1
relative fluorescence intensity^c^^,^^d^	113 ± 1, 86 ± 1	78 ± 1, 74 ± 1	61 ± 1

a UV–visible and fluorescence data were recorded in 20 mM pH 8.0 phosphate buffer solution at 15 °C.

b Maximal absorbance and extinction coefficients are reported for both electronic transitions at the wavelength of maximum absorbance (λ_max_).

c The fluorescence emission wavelength and intensity are reported for the two vibronic bands, when observed.

d Standard errors refer to the average of three independent measurements.

The absorption and emission spectra reported in Figures 7 and 8 and Table 1 were measured for the same concentration of iLOV, iLOV-Q430E, and free flavin and can therefore be compared directly. Difference spectra are shown in Figure S3 of the SI. These data indicate that the first excited state of iLOV-Q430E has a reduced extinction coefficient compared to that of iLOV. iLOV-Q430E also has a reduced fluorescence intensity (∼15–30% decrease relative to iLOV, depending on which vibronic peak is used to measure the change in intensity).

The experimental spectra indicate that the fluorescence wavelength is more sensitive to the Q430E point mutation than the absorption. This may be partially explained by comparing the excitation and emission ESTMs in Figure 4A,B, respectively. The emission ESTM indicates a higher sensitivity of the emission energy compared to the absorption. However, to determine if we can reproduce this effect in the QM/MM calculations, we repeated the ASEC-FEG calculations for both iLOV and iLOV-Q430E using the charges and gradient of the S_1_ excited state of flavin instead of the S_0_ ground state. We computed the S_1_ – S_0_ vertical emission energy at the excited-state S_1_ geometry. Note that, due to the use of excited-state charges for the flavin during the MD calculations, the protein adapts to the excited-state charge distribution. The 5 ns MD calculations have a similar time scale as a typical fluorescence lifetime, reflecting the time that the protein takes to rearrange around the excited-state configuration. In this case, it took slightly longer to achieve self-consistency of the ASEC-FEG calculations, so the first three steps were discarded and the excitation energy was averaged over the next four ASEC-FEG steps. The ASEC-FEG calculations indicate that iLOV-Q430E has a ca. 9 nm red-shifted vertical emission wavelength compared to iLOV (Table S3). Inspecting the MD simulations revealed that, on average, the E430 moves closer to the flavin in the excited state than in the ground state. The average distance between flavin’s N1 atom and the C atom of the side-chain carboxylate ion is 8.4 Å for the excited state, compared to 8.9 Å in the ground-state geometry (E430^abs^ and E430^fl^ in Figure 6). Therefore, the more significant Stokes shift in iLOV-Q430E compared to that in iLOV can be attributed to two factors. One is electronic, since the ESTM already shows a higher sensitivity of flavin’s S_0_ – S_1_ energy difference to charges near the N1 atom after flavin relaxes on its S_1_ potential energy surface to the fluorescence minimum (Figure 4). The second effect comes from the protein rearrangement; the deprotonated E430 is less repelled by the flavin excited state than the ground state and moves slightly closer, on average, to the flavin chromophore after it is excited to S_1_.

## Conclusions

Starting from simple visual guides (ESTMs), we proposed a red-shifting mutant of a flavin-binding fluorescent protein, iLOV. This prediction was further tested in this work using QM/MM ASEC-FEG calculations and, ultimately, through proof-of-principle mutagenesis and spectroscopy experiments that confirmed that the intended red shift did occur. The strategy used here, placing a negatively charged residue near flavin’s N1 atom, is an alternative to the more widely attempted and studied approach of placing a positive charge near flavin’s C4a and N5 atoms. We note that the two strategies are not mutually exclusive and may be combined to potentially achieve a further red shift of FbFPs. The calculations also indicate that further red shift may be possible by introducing a negatively charged side chain closer to the flavin N1 than in iLOV-Q430E. This could be achieved by engineering double or triple mutants that stabilize the negatively charged E430 near flavin’s N1 atom, as done for Q489 K near C4a/N5 atoms. Given the difficulties associated with stabilizing a negative charge close to the flavin N1 or with introducing two charged residues in the protein active site, we will leave such challenges to future protein engineering efforts.

This work also illustrates how computational tools and experiments can synergistically achieve a certain desired protein engineering goal. There have been multiple attempts to red shift the absorption spectrum of iLOV over the past decade. Early screening experimental studies generated tens of mutations but did not achieve the desired red shift without aid from rational design. Computational studies subsequently provided valuable insight into a strategy for how to red shift the absorption wavelength of iLOV; however, computations may miss nuances associated with point mutations that experiments can reveal. It was only through an iterative computational and experimental process that a red shift was ultimately achieved in iLOV and CagFbFP.^23,46^ In this study, we first employed simple computational tools such as ESTMs as “hypothesis generators.” We then constructed more realistic QM/MM ASEC-FEG calculations to model the proposed system and study its dynamics and spectral properties. We finally carried out the experiments to verify the results of the calculations. Conversely, the experiments often bring up new observations and questions for the calculations to answer, as was the case here for the more significant Stokes shift observed in iLOV-Q430E than in iLOV.

## Supporting Information Available

ASEC QM/MM xyz files and OpenMolcas CASPT2 output files (ZIP)
Tables of computed CASPT2//CASSCF excitation energies for iLOV and iLOV-Q430E; CASPT2//CASSCF excitation energies for lumiflavin *in vacuo* with different active spaces; and iLOV and iLOV-Q430E minus free FMN difference excitation and emission spectra (PDF)
