# Supplementary material for: Alternative Strategy for Spectral Tuning of Flavin-Binding Fluorescent Proteins
Source: J Phys Chem B. 2023 Feb 6;127(6):1301–11. doi: 10.1021/acs.jpcb.2c06475 (PMC9940217; doi:10.1021/acs.jpcb.2c06475)
Supplement: Supplementary file 2 — jp2c06475_si_002.pdf [file jp2c06475_si_002.pdf]

# Supporting information for:

# An Alternative Strategy for Spectral Tuning of Flavin-binding Fluorescent Proteins

Mohammad Pabel Kabir,[1] Daniel Ouedraogo,[1] Yoelvis Orozco-Gonzalez,[1,*] Giovanni Gadda,[1,2,3*] Samer Gozem[1,*]

[1]Department of Chemistry, Georgia State University, Atlanta, GA 30302, United States.

[2]Department of Biology, Georgia State University, Atlanta, GA 30302, United States.

[3]The Center for Diagnostics and Therapeutics, Georgia State University, Atlanta, GA 30302, United States.

## Table of content

**Table S1:** Computed vertical excitation energies for iLOV and iLOV-Q430E.

**Table S2:** Computed vertical excitation energies for iLOV and iLOV-Q430E after including 4 additional NaCl ions in the simulation.

**Table S3:** Computed vertical emission energies for iLOV and iLOV-Q430E at the excited-state minimum geometry.

**Figure S1:** CASPT2//CASSCF excitation energies for lumiflavin with different active spaces.

**Figure S2:** The 10 orbitals included in the CASPT2//CASSCF active space.

**Figure S3:** iLOV and iLOV-Q430E minus free FMN difference excitation and emission spectra.

**Table S1:** CASPT2//CASSCF/ANO-L-VDZP computed ground ($S_0$) and first singlet excited ($S_1$) state energies. Each column refers to a step of the ASEC-FEG protocol. In addition to absolute energies (in Hartree), the $S_0$-$S_1$ vertical excitation energies (in eV and nm) are also shown, as well as the shift (in nm) caused by the Q430E mutation.

| | | Step 1 | Step 2 | Step 3 | Step 4 | **Average** |
|---|---|---|---|---|---|---|
| iLOV | $S_0$ energy (Hartree) | -870.7066891 | -870.7208093 | -870.7258861 | -870.7170955 | |
| | $S_1$ energy (Hartree) | -870.5992511 | -870.6136004 | -870.6183912 | -870.6093119 | |
| | $\Delta E_{S1\text{-}S0}$ (eV and nm) | 2.924 eV (424 nm) | 2.917 eV (425 nm) | 2.925 eV (424 nm) | 2.933 eV (423 nm) | **2.925 eV (424 nm)** |
| iLOV-Q430E | $S_0$ energy (Hartree) | -870.6976046 | -870.6984876 | -870.6965667 | -870.6927712 | |
| | $S_1$ energy (Hartree) | -870.5911651 | -870.5919402 | -870.590109 | -870.5862978 | |
| | $\Delta E_{S1\text{-}S0}$ (eV and nm) | 2.896 eV (428 nm) | 2.899 eV (428 nm) | 2.897 eV (428 nm) | 2.897 eV (428 nm) | **2.897 eV (428 nm)** |
| Shift caused by the Q430E mutation | $\Delta\Delta E_{S1\text{-}S0}$ (nm) | | | | | **4 nm** |

**Table S2:** CASPT2//CASSCF/ANO-L-VDZP computed ground ($S_0$) and first singlet excited ($S_1$) state energies in the presence of 4 additional $Na^+$ and $Cl^-$ ions. Each column refers to a step of the ASEC-FEG protocol. In addition to absolute energies (in Hartree), the $S_0$-$S_1$ vertical excitation energies (in eV and nm) are also shown, as well as the shift (in nm) caused by the Q430E mutation.

| | | Step 1 | Step 2 | Step 3 | Step 4 | **Average** |
|---|---|---|---|---|---|---|
| iLOV | $S_0$ energy (Hartree) | -870.7303668 | -870.7269008 | -870.7253756 | -870.708917 | |
| | $S_1$ energy (Hartree) | -870.623182 | -870.6190768 | -870.6190198 | -870.602247 | |
| | $\Delta E_{S1\text{-}S0}$ (eV and nm) | 2.917 eV (425 nm) | 2.934 eV (423 nm) | 2.894 eV (428 nm) | 2.903 eV (427 nm) | **2.912 eV (426 nm)** |
| iLOV-Q430E | $S_0$ energy (Hartree) | -870.7099589 | -870.7178432 | -870.7119662 | -870.6988243 | |
| | $S_1$ energy (Hartree) | -870.603197 | -870.6106224 | -870.605017 | -870.5911968 | |
| | $\Delta E_{S1\text{-}S0}$ (eV and nm) | 2.905 eV (427 nm) | 2.918 eV (425 nm) | 2.910 eV (426 nm) | 2.929 eV (423 nm) | **2.915 eV (425 nm)** |
| Shift caused by the Q430E mutation | $\Delta\Delta E_{S1\text{-}S0}$ (nm) | | | | | **0 nm** |

**Table S3:** CASPT2//CASSCF/ANO-L-VDZP computed ground ($S_0$) and first singlet excited ($S_1$) state energies at the $S_1$ minimum geometry. Each column refers to a step of the ASEC-FEG protocol. Note that the calculations needed 3 steps to reach self-consistency, so results from Steps 1-3 were discarded. In addition to absolute energies (in Hartree), the $S_0$-$S_1$ vertical excitation energies (in eV and nm) are also shown, as well as the shift (in nm) caused by the Q430E mutation.

| | | Step 4 | Step 5 | Step 6 | Step 7 | **Average** |
|---|---|---|---|---|---|---|
| iLOV | $S_0$ energy (Hartree) | -870.7135153 | -870.7162409 | -870.7212094 | -870.7294337 | |
| | $S_1$ energy (Hartree) | -870.6324525 | -870.6344888 | -870.6396116 | -870.6477739 | |
| | $\Delta E_{S1\text{-}S0}$ (eV and nm) | 2.206 eV (562 nm) | 2.225 eV (557 nm) | 2.220 eV (558 nm) | 2.222 eV (558 nm) | **2.218 eV (559 nm)** |
| iLOV-Q430E | $S_0$ energy (Hartree) | -870.6925643 | -870.6866233 | -870.7039868 | -870.6853863 | |
| | $S_1$ energy (Hartree) | -870.6124873 | -870.6063316 | -870.6236473 | -870.6050131 | |
| | $\Delta E_{S1\text{-}S0}$ (eV and nm) | 2.179 eV (569 nm) | 2.185 eV (567 nm) | 2.186 eV (567 nm) | 2.187 eV (567 nm) | **2.184 eV (568 nm)** |
| Shift caused by the Q430E mutation | $\Delta\Delta E_{S1\text{-}S0}$ (nm) | | | | | **9 nm** |

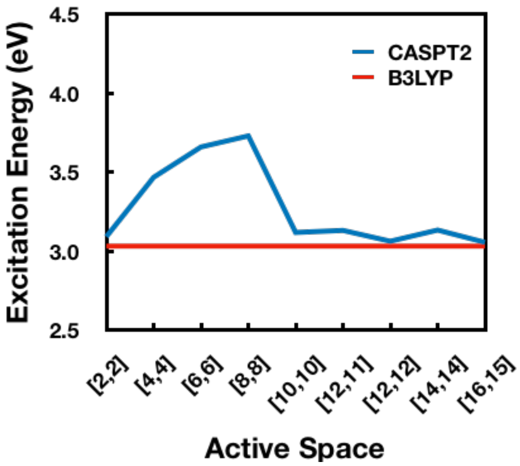


**Figure S1:** A plot of the $S_0$-$S_1$ excitation energy vs active space for CASPT2/ANO-L-VDZP calculations for an MP2-optimized gas-phase geometry of lumiflavin. Only π and π* orbitals were included in the active space in all cases. The first calculation was performed using a [16,15] active space, and the active space was then reduced gradually by removing orbitals based on their occupancies. The calculations indicate that there is a limited benefit to going beyond a [10,10] active space for vertical excitation energies. The TD-DFT/cc-pVTZ excitation energy is added as a reference in red because it was found to reproduce the experimental spectrum of FMN in solution accurately when accounting for Franck-Condon factors (Kabir, M.P. et al., PCCP, 2019, *21*, 16526-16537).

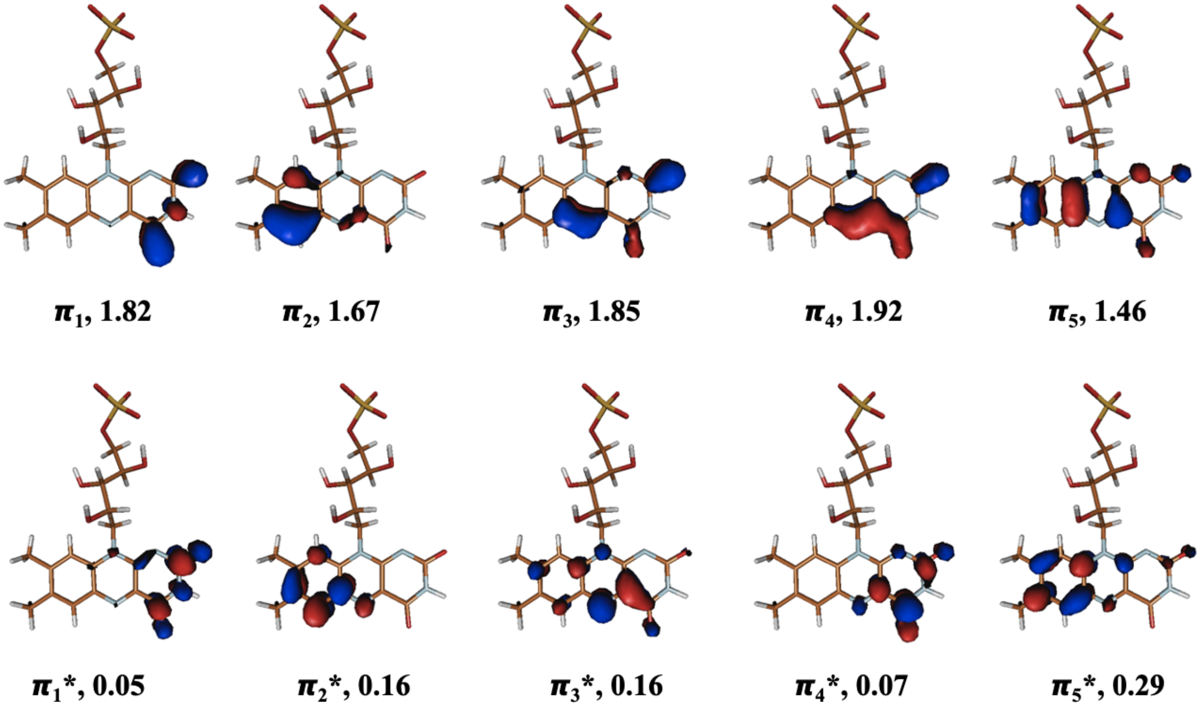


**Figure S2:** The 10 orbitals included in the CASPT2//CASSCF active space.

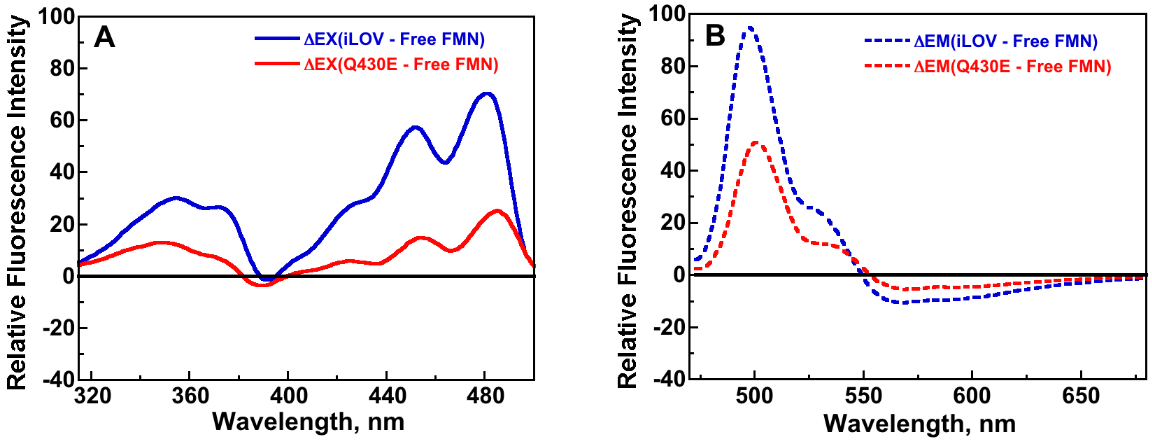


**Figure S3: A.** Difference excitation spectra of iLOV and iLOV-Q430E minus that of free FMN. **B.** Difference emission spectra of the iLOV and iLOV-Q430E minus that of free FMN. The reference line in panels A and B represents the ΔEX (free FMN-Free FMN) and ΔEM (free FMN-Free FMN), respectively.
